# Bioinformatic strategies in metagenomics of chronic prostatitis

**DOI:** 10.1007/s00345-025-05514-7

**Published:** 2025-03-26

**Authors:** Elmira Davasaz Tabrizi, Mushteba Sevil, Ercan Arican

**Affiliations:** 1 Department of Molecular Biology and Genetic, Istanbul University, Istanbul, Turkey; 2 Department of Surgery and Urology, Medicalpark Hospital, Izmir, Turkey

**Keywords:** Chronic prostatitis, Metagenomic, Bioinformatic, Urinary microbiome, Antibiotic resistance genes

## Abstract

**Purpose:**

Chronic prostatitis/chronic pelvic pain syndrome (CP/CPPS) is a prevalent urological condition in young men, significantly affecting quality of life due to persistent discomfort and neuropsychological symptoms. Despite its high prevalence, the etiology of CP/CPPS remains poorly understood. This study investigated urinary microbiota differences between CP/CPPS patients and healthy controls to identify microbial contributors, antibiotic resistance genes (ARGs), and virulence factors of dominant bacteria, as well as to explore potential therapeutic targets.

**Methods:**

Urine samples were collected from 58 CP/CPPS patients and 25 controls. Symptom severity was assessed by a specialist urologist using the NIH Chronic Prostatitis Symptom Index and UPOINT classification. Bacterial-specific 16 S rRNA sequencing was performed using nanopore technology, with bioinformatics analyses conducted via ONT guppy 5.0.11, NCBI and SLV 16 S bacterial taxonomic databases, UPGMA hierarchical clustering, and the Bacterial and Viral Bioinformatics Resource Center (BV-BRC). Pairwise comparisons were analyzed using the Mann-Whitney U test.

**Results:**

Distinct microbial diversity patterns were observed between patients and controls. *Bacillus* species were significantly enriched in CP/CPPS patients, while *Enterococcus* species predominated in controls. Younger patients exhibited unique microbiome profiles compared to older groups. Bioinformatics analyses identified ARGs and virulence factors associated with *Bacillus* species, implicating them in localized inflammation. Antibiotics like pleuromutilin or vancomycin were identified as potential therapeutic options, though experimental validation was beyond the study’s scope.

**Conclusion:**

These findings highlight microbial imbalances and provide a foundation for microbiome-targeted therapeutic strategies for CP/CPPS management in the future. Additionally, the identification of bacterial virulence factors and ARG provides insights into the potential mechanisms driving persistent symptoms. Future research with larger cohorts and experimental validation of the suggested therapeutic options may contribute to more effective treatment for CP/CPPS.

**Supplementary Information:**

The online version contains supplementary material available at 10.1007/s00345-025-05514-7.

## Introduction

**Fig. 1:**
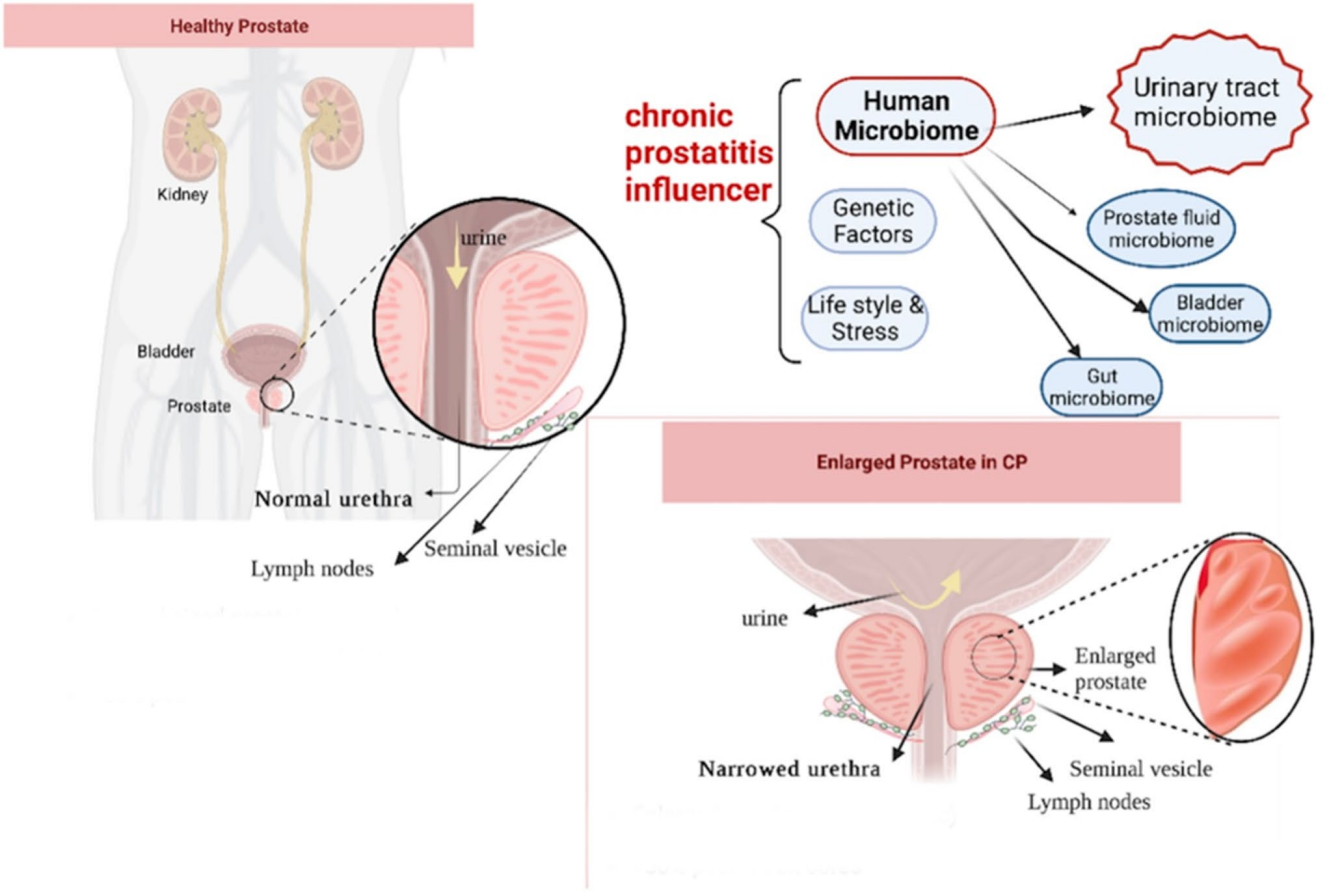
Summary of Etiological Factors Contributing to Chronic Prostatitis/Chronic Pelvic Pain Syndrome (CP/CPPS). Prostate anatomy and its potential role in Chronic prostatitis, or chronic pelvic pain syndrome (CP/CPPS) pathophysiology. While significant prostate enlargement is uncommon in CPPS, mild enlargement may contribute to urethral narrowing, leading to urinary retention and incomplete bladder emptying. This can create an environment conducive to bacterial persistence and infection, potentially exacerbating CPPS symptoms. Studies have shown that prostate inflammation plays a role in CPPS pathogenesis, with evidence of inflammatory cytokines and ERK1/2 signaling activation in CPPS patients [7]. Understanding these anatomical and inflammatory changes provides context for the microbial and immune dysregulation observed in CPPS. CP/CPPS is influenced by various factors, including lifestyle, genetics, and notably, the human microbiome. The microbiota in prostate fluid, bladder, gut, and other components of the urinary tract plays a critical role in the pathogenesis of this chronic condition. Understanding how microbial communities interact and contribute to the development and persistence of CP/CPPS remains an important area of research, offering insights into potential therapeutic targets and interventions for affected individuals

Chronic prostatitis/chronic pelvic pain syndrome (CP/CPPS), classified as category III prostatitis, is a complex condition that profoundly affects patient quality of life [1, 2]. Despite extensive research, the underlying mechanisms of CP/CPPS remain poorly understood, and therapeutic options are limited. Current treatment modalities, including physiotherapy, alpha-blockers, and psychological interventions, have demonstrated variable success rates [3, 4]. The longstanding hypothesis that a “hidden” pathogen undetectable by traditional culturing methods is responsible has driven the overuse of antibiotics. This practice has contributed to the alarming rise in antimicrobial resistance, further complicating treatment and imposing a significant burden on both patients and healthcare systems [5, 6] (Fig. 1).

This study is clinically significant because CP/CPPS represents a major challenge in urology, with unmet needs for effective and targeted therapies. Understanding the microbial imbalances and antibiotic resistance gene (ARG) profiles of dominant bacteria associated with CP/CPPS could lead to more precise and personalized treatment strategies. Specifically, identifying bacterial populations, such as the dominance of Bacillus in CP/CPPS patients, and characterizing their resistance patterns provides valuable insights into potential therapeutic targets. These findings aim to inform antibiotic selection, reduce recurrence, and improve patient outcomes.

The advent of high-throughput sequencing technologies, particularly 16 S rRNA sequencing, has revolutionized the study of microbial diversity in non-culturable conditions [8]. Nanopore sequencing platforms, such as MinION, enable real-time assembly of complete microbial genomes with high accuracy and longer reads, offering significant advantages over traditional short-read sequencing methods [6]. These technological advancements have opened new avenues for exploring the etiology of CP/CPPS, a disease where microbial contributors and their functional roles remain elusive.

Our hypothesis posits that microbial imbalances, coupled with distinct ARG profiles, play a critical role in the pathogenesis of CP/CPPS. We further hypothesize that these imbalances contribute to localized inflammation and symptom severity. This study investigates whether ARGs and virulence factors in dominant bacterial species, such as *Bacillus velezensis*, can serve as biomarkers for targeted treatment approaches. By profiling ARGs and analyzing microbial communities, we aim to identify therapeutic options that reduce inflammation and optimize antibiotic selection. This research is among the first to utilize nanopore-based metagenomic sequencing to investigate microbial communities in CP/CPPS patients across different age groups. Notably, to our knowledge, we identified *Bacillus velezensis* as a potential pathogenic influencer, marking its first association with CP/CPPS. Comparative genome analysis revealed that *B. velezensis* possesses a competitive advantage through the production of secondary metabolites with antibiotic-like properties, positioning it as a critical target for future research into its role in chronic inflammation and its potential as a drug target [9, 10]. While these bioinformatics tools provided robust analyses, revealing antibiotics such as vancomycin and pleuromutilin as potential options, experimental and clinical validation of resistance mechanisms was beyond the scope of this study.

Furthermore, our study is one of the few to compare bacterial communities at the species level in CP/CPPS, leveraging cutting-edge bioinformatics tools like BV-BRC and Galaxy [11, 12]. These platforms facilitated a comprehensive analysis of microbial metabolic pathways and virulence factors, offering actionable insights into targeted therapeutic development. These findings may support future drug licensing efforts and guide the development of precise therapeutic approaches for CP/CPPS, addressing a critical unmet need in clinical practice.

## Experimental Procedure

### Sample collection and DNA extraction

Urine (midstream) samples were collected from patients referred to Bilge Hospital’s urology department. The sample collection followed sterile protocols, using the clean-catch method, consistent with practices in urological microbiome studies [13, 14]. Symptomatic and asymptomatic individuals were identified by an experienced specialist urologist (surgeon) based on the NIH Category III criteria [2]. Symptomatic patients who had not received antibiotics within the preceding three months were included in the study, with asymptomatic individuals serving as controls under the same antibiotic-free conditions. The inclusion criteria targeted CP/CPPS patients specifically, while exclusion criteria eliminated individuals with comorbidities such as organ transplants, immunosuppressive therapy, BPH (Benign prostatic hyperplasia), or diabetes. Age groups included Group 1 (29–39 years), Group 2 (40–49 years), and Group 3 (50 + years), with controls from all age groups. Samples were centrifuged, and DNA extraction was performed using the QIAamp DNA Micro Kit and Hibrigen Urine DNA Isolation Kit with modified protocols [15, 16].

### 16 S-rRNA gene library preparation and sequencing processing

Extracted DNA was used to create 16 S PCR amplicons for each age group, adhering to the Oxford Nanopore 16S024 kit primers. Sequencing was performed on the MinION platform (ONT FLO-MIN106D), a technology known for high-throughput, real-time sequencing, ideal for diverse microbial communities [17, 18].

### Multi-layered technological strategies in metagenomics and microbial biotechnology

This study integrated advanced technological methods, utilizing the MinION Nanopore platform for long-read sequencing, allowing for more comprehensive microbial identification in chronic prostatitis samples. This platform’s real-time sequencing capabilities, combined with bioinformatic pipelines such as Galaxy and the BV-BRC, provided high-resolution insights into microbial compositions and potential pathogens, including *Bacillus velezensis* as a novel influencer [12]. Taxonomic classification and antibiotic resistance gene (ARG) profiling were conducted using the BV-BRC platform, which employs the Kraken 2 taxonomic sequence classification system. Kraken 2 improves upon its predecessor by using k-mer matching, minimizers, and spaced seeds, enhancing classification speed and accuracy while reducing database size. The study utilized a comprehensive database integrating 16 S sequences from NCBI’s RefSeq, GenBank, and SILVA for robust microbial identification.

To ensure the reliability of taxonomic assignments, BV-BRC results were cross-validated with outputs from NCBI and SILVA, confirming consistent microbial classifications, including *Bacillus velezensis*, the dominant species in CP/CPPS patients. This multi-database approach reinforced the robustness of the analyses, minimizing potential classification errors and increasing confidence in the study’s microbiome profiling results.

### Computational biology approaches to ARG and VF

In this study, we leveraged computational tools to investigate the prevalence of antibiotic resistance genes (ARGs) and virulence factors (VFs) in dominant bacterial species from chronic prostatitis samples, using the BV-BRC database. ARG and VF data were obtained from the genomes of five dominant bacterial species identified in the patient groups, alongside one key species detected in healthy control samples. Despite the detection of ARGs within these genomes, the mere presence of these genes does not always correlate with an antibiotic-resistant phenotype. Special consideration must be given to the role of single nucleotide polymorphisms (SNPs) in resistance mechanisms, as these mutations can influence the induction of resistance pathways. To further illustrate how single nucleotide polymorphisms (SNPs) can contribute to antibiotic resistance, examples from *Escherichia coli* and *Mycobacterium tuberculosis* highlight the impact of specific mutations in key genes. In *E. coli*, mutations in the *gyrA* gene—part of the DNA gyrase complex—have been associated with fluoroquinolone resistance, affecting the drug’s binding efficiency. For *M. tuberculosis*, SNPs in the *katG* and *rpoB* genes result in resistance to isoniazid and rifampicin, respectively. These SNPs alter either the drug-binding sites or the target proteins, promoting resistance without the necessity of additional resistance genes [19, 20].

The data were processed through the Usegalaxy platform, which enabled comprehensive bioinformatic analyses. Heat maps and circos plots were generated to visualize the distribution of ARGs and VFs across the species (Figs. 3, 4 and 5). These computational approaches are crucial for high-resolution pathogen profiling, enabling the identification of resistance genes and virulence factors that may contribute to chronic prostatitis pathogenesis. Multi-layered analyses help to elucidate bacterial interactions and adaptive mechanisms, offering insights into microbial contributions to disease progression. Such findings are instrumental for future diagnostic or therapeutic strategies targeting chronic prostatitis [21]. Additionally, this method allows for the discovery of novel interactions between bacteria and their host, which could influence both treatment options and the design of precision medicine interventions [22].

**Fig. 2:**
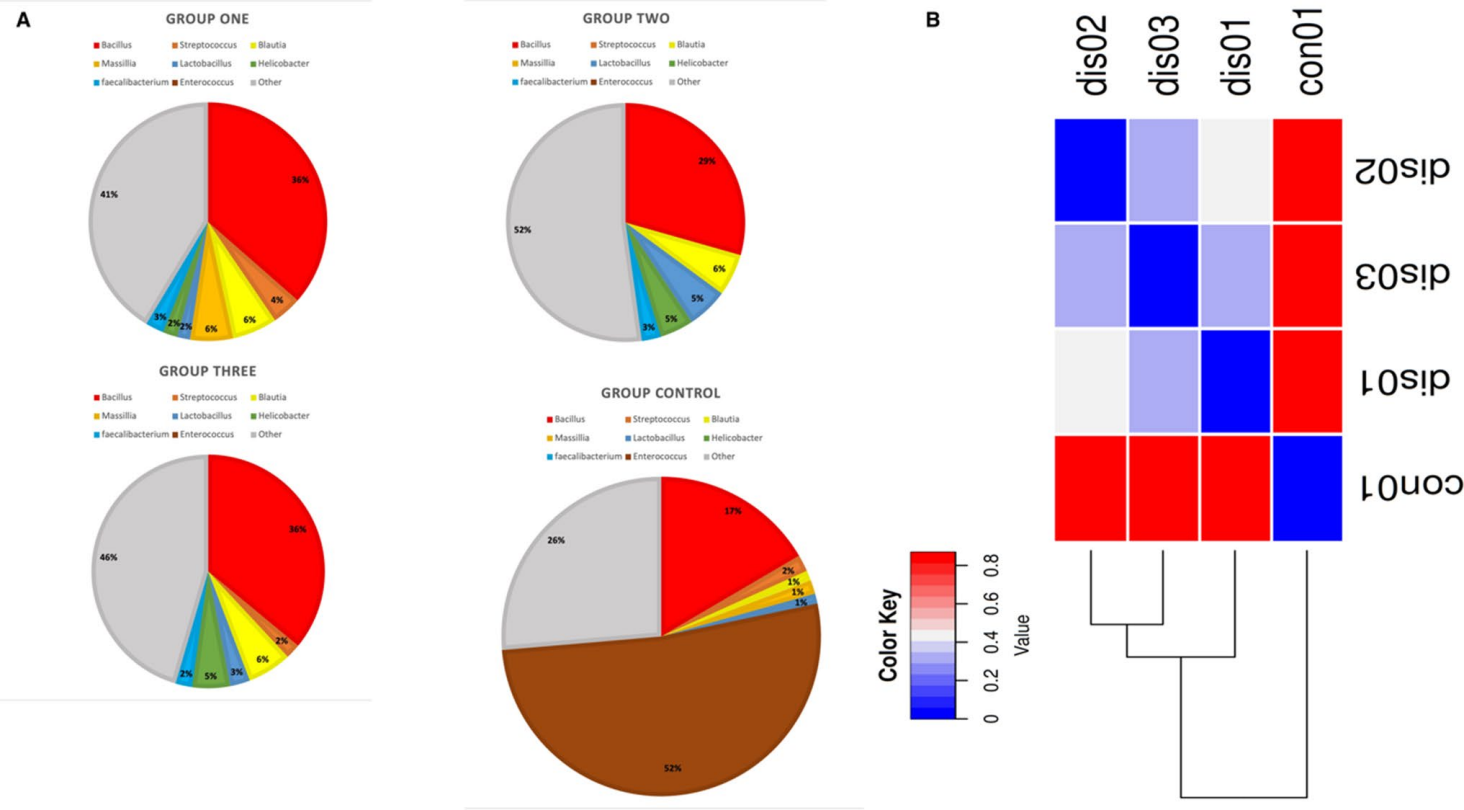
Bacterial Distribution and UPGMA Dendrogram for Chronic Prostatitis/Chronic Pelvic Pain Syndrome (CP/CPPS) Patients and Healthy Controls. (**A**) Taxonomic distribution of microbiota in CP/CPPS patients (*n* = 58; subgroup 1: *n* = 23, subgroup 2: *n* = 20, subgroup 3: *n* = 15) and healthy controls (*n* = 25), using the BV-BRC database as the primary reference. The highest microbial diversity was observed in the youngest patient subgroup (29–39 years). The Bacillus genus (red) was the dominant bacterial group in the patient cohort, while Enterococcus (brown) was the most abundant genus in the healthy controls. Other bacterial genera, including Blautia, Faecalibacterium, and Helicobacter, were significantly reduced in abundance in the patient group compared to the controls. (**B**) Beta-diversity heatmap and UPGMA dendrogram based on Bray-Curtis dissimilarity index, demonstrating distinct clustering patterns. Healthy controls formed a well-defined separate cluster, while patient subgroups 1 and 3 exhibited closer microbial composition and clustering. Subgroup 2 showed a little separation from both the other patient subgroups. Statistical analyses confirmed significant differences in microbial composition between healthy and patient groups, highlighting the unique microbial shifts associated with CP/CPPS

**Fig. 3:**
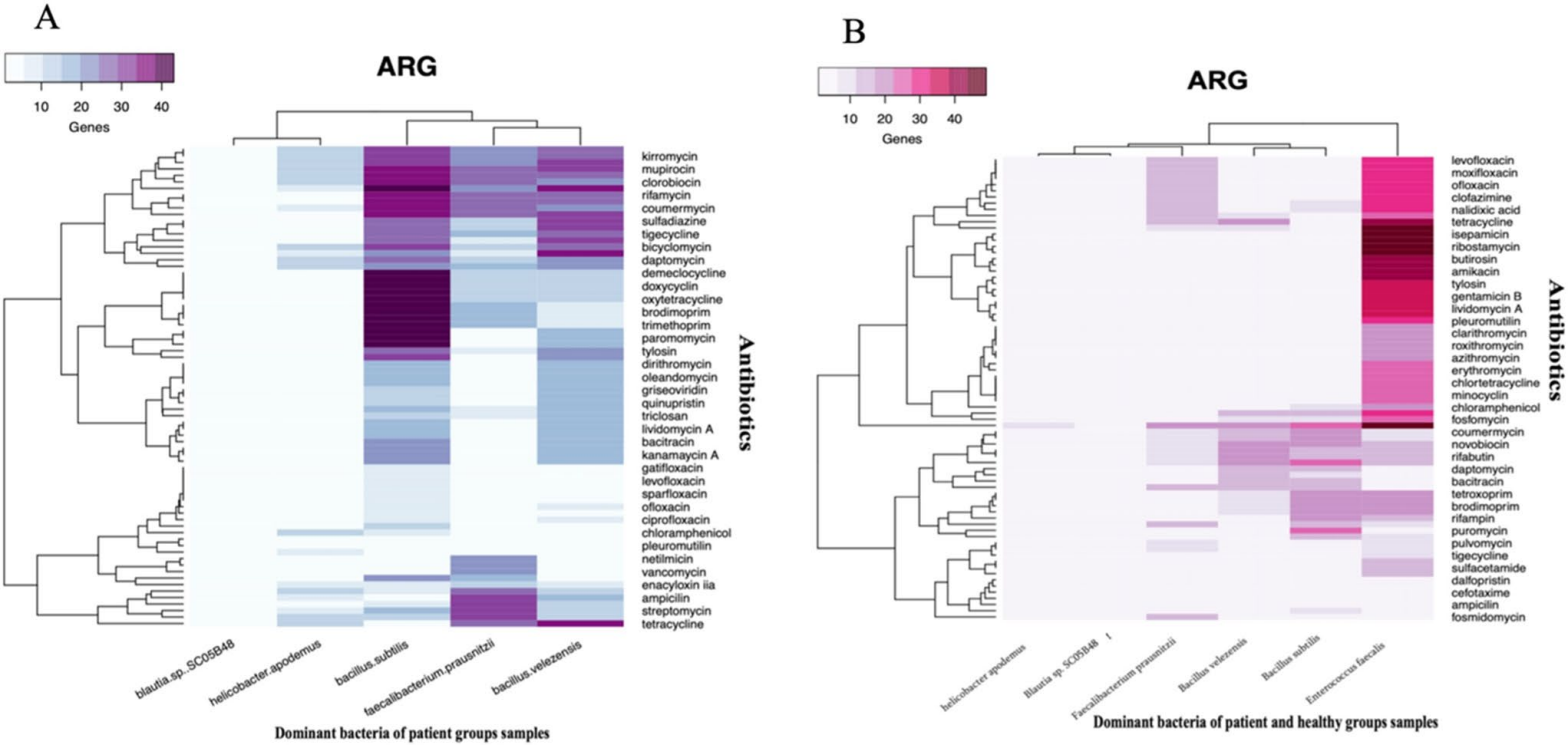
Antibiotic Resistance Gene (ARG) Profiles in Key Bacterial Genera. (**A**) Heatmap illustrating the abundance of ARGs across dominant bacterial species identified in patient samples. The Bacillus genus exhibits a notably high number of ARGs, with Bacillus subtilis carrying the largest resistance gene burden, followed by Bacillus velezensis, Faecalibacterium, and Helicobacter. In contrast, Blautia harbors the lowest number of resistance genes. The data presented reflects ARGs identified in bacterial genome structures available in the BV-BRC database as of 2024. (**B**) Heatmap comparing ARG profiles of dominant bacterial genera in both patient and healthy groups, with a specific focus on Enterococcus faecalis. E. faecalis exhibits a significantly greater diversity and abundance of ARGs compared to bacterial species from patient samples. Among the antibiotic classes analyzed, E. faecalis displays notably high resistance. As highlighted in previous research discussed in the manuscript, pleuromutilins—considered a potential antibiotic option—also show a high number of resistance genes in E. faecalis according to our analysis. In contrast, Bacillus species carry significantly fewer resistance genes to pleuromutilins, underscoring potential therapeutic option. Point: Antibiotics were selected based on the ARG burden within dominant bacterial genera. Note: The antibiotic classes displayed in the two heatmaps differ due to E. faecalis’ genome carrying resistance genes to a wider range of antibiotics. The study emphasizes key antibiotic classes for each dominant bacterial genus, providing a basis for future research to validate their clinical relevance. These findings are based on bioinformatics analyses of genome data available in the BV-BRC database as of 2024. The clinical validity of these results requires further experimental studies and validation

**Fig. 4:**
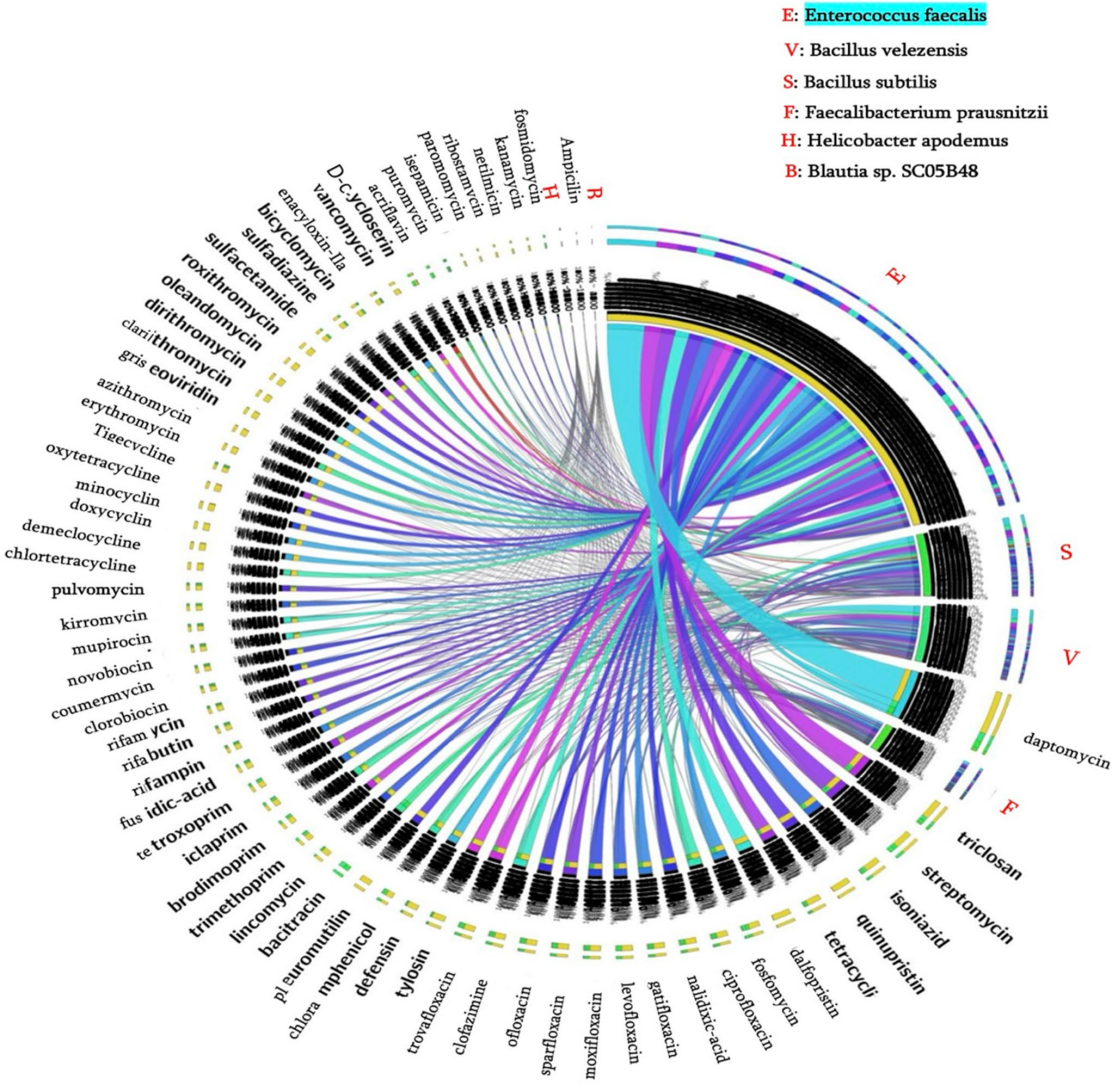
Circos Plot of Antibiotic Resistance Gene (ARG) Profiles in Dominant Bacterial Species Across Healthy and Patient Groups. This Circos plot depicts the distribution of antibiotic resistance genes (ARGs) across six dominant bacterial species—Enterococcus faecalis, Bacillus velezensis, Bacillus subtilis, Faecalibacterium prausnitzii, Helicobacter apodemus, and Blautia sp. SC05B48—in healthy controls and CP/CPPS patients. The plot highlights resistance patterns against 65 antibiotics. Enterococcus faecalis, dominant in the healthy group, occupies the largest segment of the circle, reflecting its high ARG burden compared to the other species. Bacillus velezensis and Bacillus subtilis, predominantly found in the patient group, follow E. faecalis in carrying a significant number of resistance genes. All three species (E. faecalis, B. velezensis, and B. subtilis) show high resistance to daptomycin, eliminating it as a viable treatment option in the first stage of antibiotic selection. In contrast, Faecalibacterium prausnitzii, Helicobacter apodemus, and Blautia sp. SC05B48 demonstrate the lowest ARG burden, with Blautia sp. SC05B48 exhibiting the fewest resistance genes overall. Triclosan emerges as a promising therapeutic option, as B. velezensis and B. subtilis are sensitive to it, despite E. faecalis carrying resistance genes against this antibiotic. Limitations: The data are derived from the genomic analysis of these bacterial species as recorded in the BV-BRC database (up to 2024). As new ARGs are identified, the findings may evolve. These results are based on computational predictions and require further clinical validation to confirm their correlation with actual resistance profiles and therapeutic outcomes

**Fig. 5:**
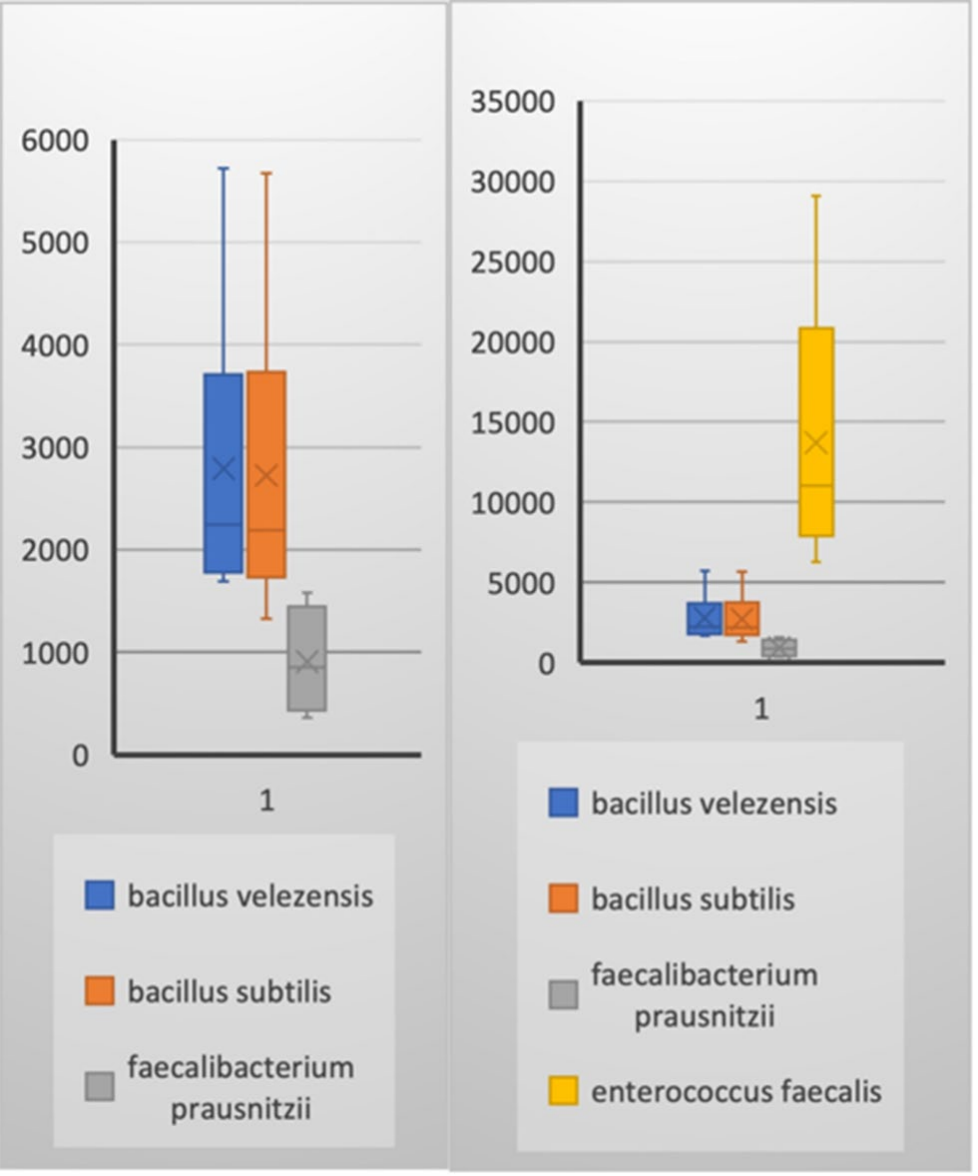
Distribution of Antibiotic Resistance Genes (ARGs) Across Dominant Bacterial Species for Five Antibiotic Classes. The frame illustrates the prevalence of ARGs associated with resistance to five different antibiotics within the dominant bacterial species isolated from patient and healthy groups. Each bar represents the number of ARGs detected for a given antibiotic class, indicating the potential resistance profile and diversity within the microbiome. ARGs for 1-daptomycin, 2-bacitracin, 3-triclosan, 4-streptomycin, 5-isoniazid, and 6-fosfomycin antibiotics in healthy and patient groups dominant bacteria species. In general, Enterococcus has a high abundance of ARGs compared with other microorganisms. Moreover, among the bacterial genera of the patient groups, Bacillus contains more ARGs than other microorganisms, such as Faecalibacterium prausnitzii

### Bioinformatic and statistical analyses

Raw 16 S rRNA data were processed using ONT’s Guppy tool (v5.0.11). Consensus sequences were generated using Bbtools (v38.91), MagicBLAST (v1.6.0), and SAMtools (v1.13) for further classification. OTU tables were constructed using multiple taxonomies such as NCBI, BV-BRC, SILVA, and RDP, following guidelines from the latest metagenomics studies [23]. All statistical analyses were performed to evaluate differences between the patient and control groups and to assess microbial composition diversity.

Pairwise Comparisons and Diversity Analysis: The Mann-Whitney U test was used for pairwise comparisons, given the non-parametric nature of the data. For beta-diversity analysis, the Bray-Curtis dissimilarity index was employed to quantify pairwise differences in microbial community composition [24, 25], incorporating both the presence/absence and relative abundance of taxa. These analyses were conducted using QIIME2 and Python (version 3.9). The robustness of clustering patterns observed in the beta-diversity analysis was assessed using permutation tests, confirming the significance of identified differences. Effect sizes (*d*) were calculated for pairwise group comparisons using Cohen’s *d*, providing a standardized measure of the magnitude of differences. Confidence intervals (*CI*s) were calculated at a 95% confidence level to evaluate differences in *Bacillus* bacterial populations across groups. Standard deviations (*SD*) for each group were estimated using the formula:

*SD*= $\:\sqrt{\frac{\rho\:(1-\rho\:)}{n}}$​​, where *p* is the mean proportion, and *n* is the sample size. The standard error (SE) for each group was computedas: *SE*= $\:\frac{SD}{\sqrt{n}}$. and the confidence intervals were derived as: *CI* = $\:\rho\:\mp\:\text{{\rm\:Z}}\cdot\:SE\:\:\:\:$with Z = 1.96 for a 95% confidencelevel.

Power analysis was conducted using Python (version 3.9) to determine the adequacy of sample sizes for detecting significant differences (1 − *β*) at a significance level of α = 0.05. Results indicated sufficient power (> 0.8) for comparisons involving the patient and healthy control groups but lower power (< 0.8) for comparisons between some patient subgroups, reflecting smaller observed differences. Statistical tests, including the Mann-Whitney U test, effect size calculations, power analysis, and *CI* estimations, are summarized in Tables 7 and 1, and 2.

**Table 1:** Effect size and Power Analysis; effect sizes (Cohen’s dd) and statistical power (1 − β) for each pairwise comparison between patient subgroups and the healthy control group. The analysis provides a standardized measure of the magnitude of differences and the adequacy of sample sizes

Comparison	Effect Size (Cohen’s d)	Power (1 − β)
Group 1 vs. Group 2	0.707	0.70
Group 1 vs. Group 3	0.00	0.05
Group 1 vs. Healthy	1.94	0.99
Group 2 vs. Group 3	0.86	0.80
Group 2 vs. Healthy	1.62	0.97
Group 3 vs. Healthy	2.57	0.99

**Table 2:** Confidence interval (CI): confidence intervals (CIs) for the relative abundance of Bacillus bacterial populations across groups. The table reports the 95% CI range for each comparison, calculated using standard error (SE) and sample size. The observed CI ranges suggest that there is no substantial overlap between the patient and healthy groups, highlighting a potential difference in bacterial populations that warrants further investigation

group	$\:\varvec{\rho\:}$	*n*	95% CI (Lower - Upper)
1 [young patient]	0.36	23	31.9 − 40%
2 [middle-aged patient]	0.29	20	24 − 34%
3 [older patients]	0.36	15	29 − 42%
Healthy controls	0.17	25	14 − 19%

**Table 3:**
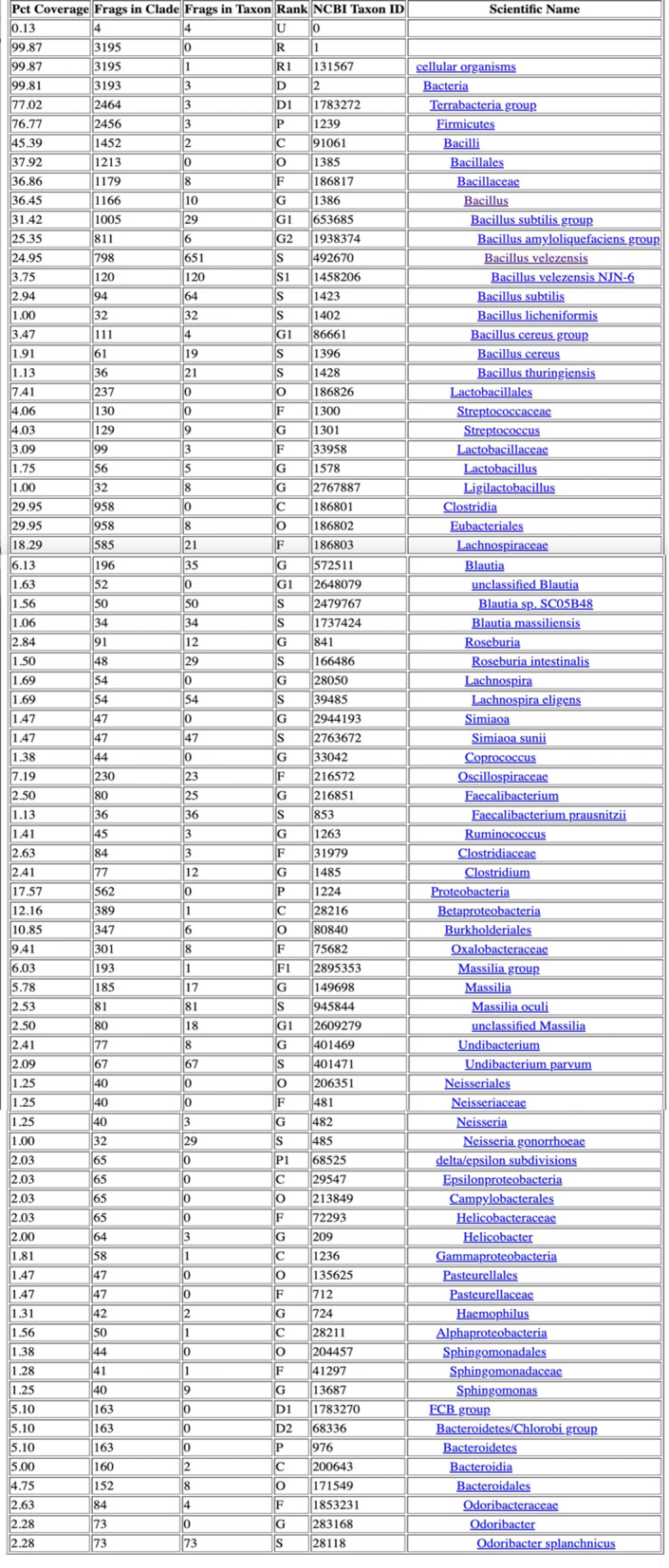
Taxonomic Classification Report for group 1. Input data: Single-end Libraries

**Table 4:**
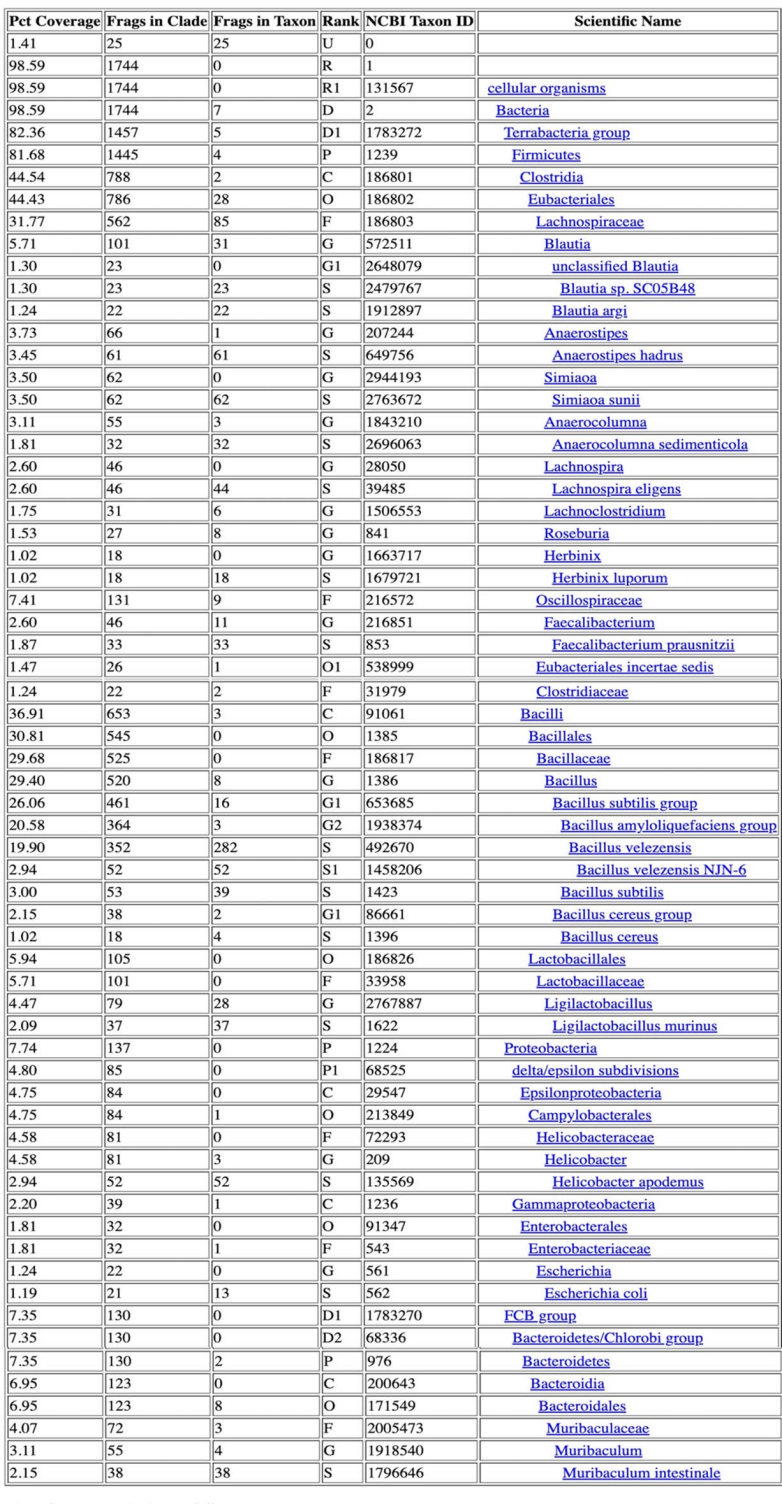
Taxonomic Classification Report for group 2. Input data: Single-end Libraries

**Table 5:**
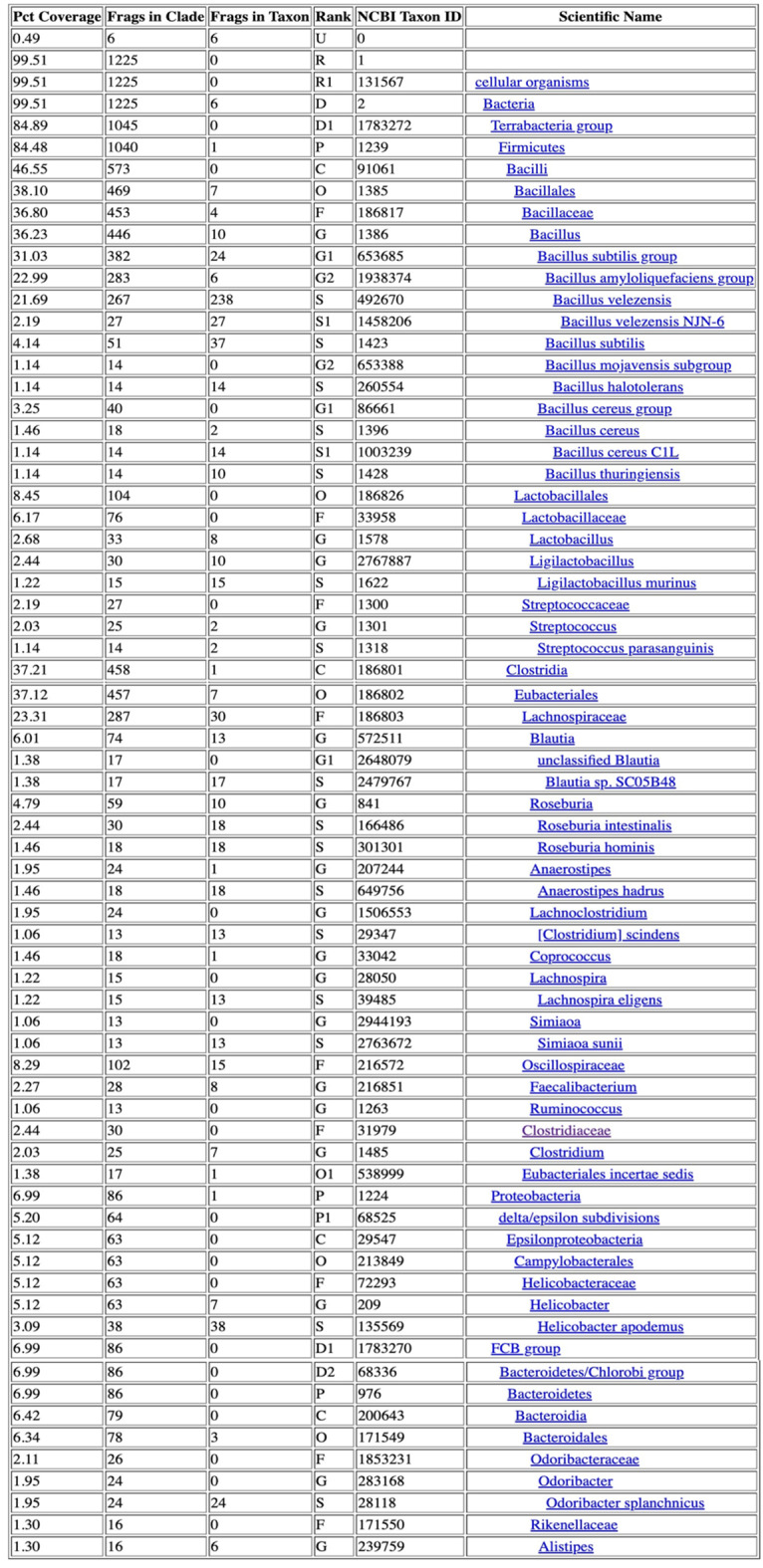
Taxonomic Classification Report for group 3. Input data: Single-end Libraries

**Table 6:**
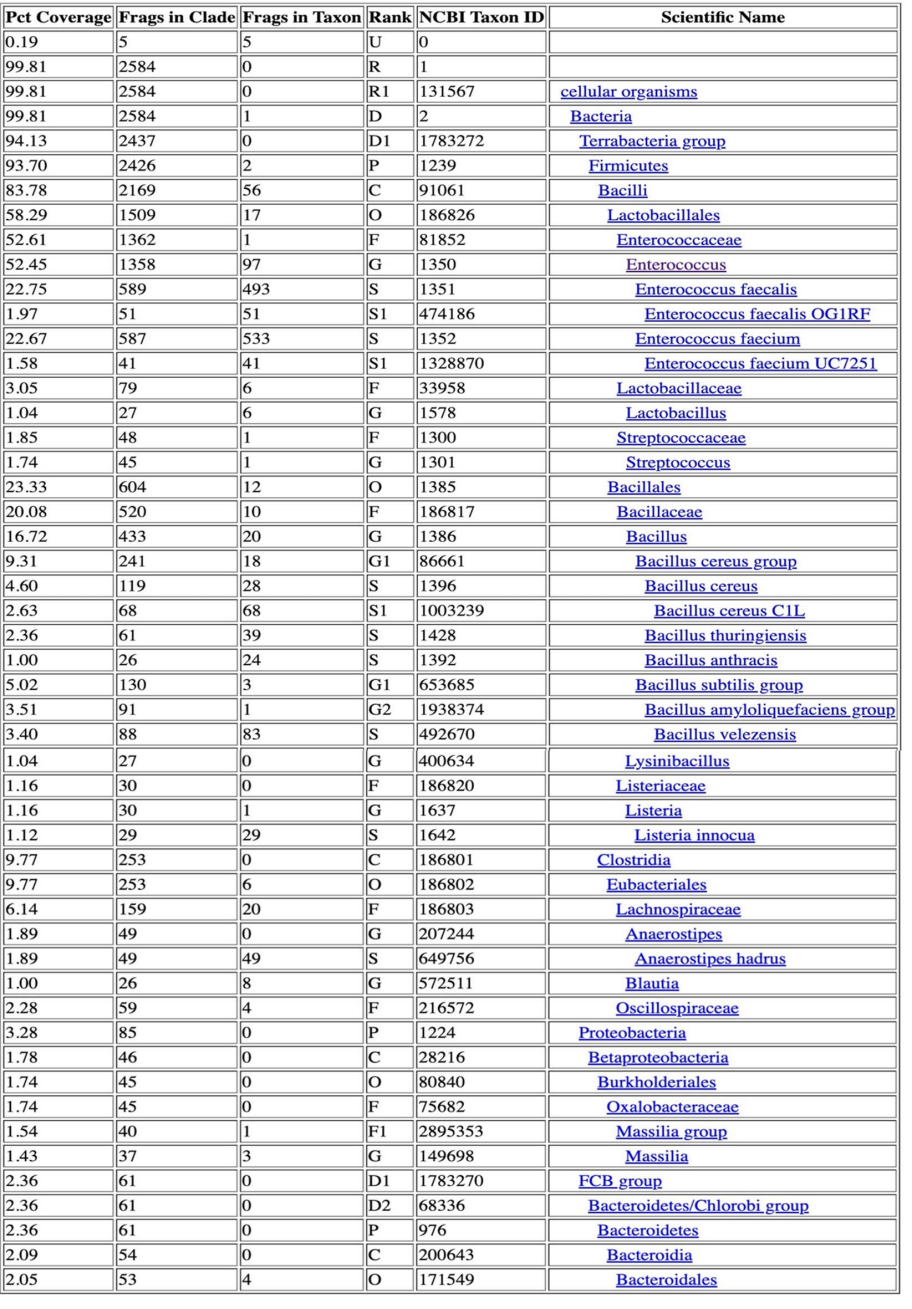
Taxonomic Classification Report for control group. Input data: Single-end Libraries

**Table 7:** Mann-Whitney U Test, Summary of the Mann-Whitney U test results for pairwise comparisons between patient subgroups and the healthy control group. The U statistic and corresponding p-values are reported, highlighting significant differences in microbial composition diversity of patients and healthy groups

Comparison	U Statistic	*p*-value	Significance
Group 1 vs. Group 2	361.0	0.0015	Significant (*p* < 0.05)
Group 1 vs. Group 3	262.0	0.0270	Significant (*p* < 0.05)
Group 1 vs. Healthy	556.0	3.19 × 10^− 8^	Highly significant (*p* < 0.01)
Group 2 vs. Group 3	117.0	0.1760	Not significant (*p* > 0.05)
Group 2 vs. Healthy	422.0	0.0001	Significant (*p* < 0.05)
Group 3 vs. Healthy	383.0	1.07 × 10^− 6^	Highly significant (*p* < 0.01)

### Ethics statement

The study protocol was reviewed and approved by the Clinical Research Ethics Committee of Istanbul University Medical Faculty (Approval No: 910, Letter No: 108788). Informed consent was obtained from all participants.

## Results

This study included a total of 83 participants, with 58 samples obtained from patients experiencing chronic prostatitis and 25 from asymptomatic healthy individuals as a control group. Among the patient cohorts, the youngest age group (29–39 years) exhibited the highest microbial diversity, suggesting that age may play a role in microbiome composition and its potential association with prostatitis symptoms. Microbial diversity and taxonomic distribution were assessed across five major bioinformatics databases, with the Bacterial & Viral Bioinformatics Resource Center (BV-BRC) results (Tables 3, 4, 5 and 6) serving as the primary reference for creating comprehensive visualizations and statistical analyses (Fig. 2A). These findings underline the variability in microbial profiles across age groups, supporting the potential role of microbiome diversity in chronic prostatitis pathology.

The Shapiro-Wilk test for normality indicated that the dataset did not follow a normal distribution (*p* < 0.05). Consequently, we applied nonparametric tests, specifically with a 95% confidence level. The Mann-Whitney U test identified bacterial taxa with significant compositional differences between control and patient groups, particularly highlighting points where microbial profiles diverged most substantially (Table 7). Additionally, Effect size analysis (Cohen’s d**)** demonstrated strong effect sizes (d > 1.5, power > 0.95) for major bacterial differences, particularly in *Bacillus* abundance in CP/CPPS patients versus controls. Confidence Interval (CI) analysis further confirmed that Bacillus abundance was significantly higher in patients, while *Enterococcus* remained dominant in controls, reinforcing the robustness of microbial composition differences.

To further investigate community diversity, we employed a beta-diversity heatmap and an Unweighted Pair Group Method with Arithmetic Mean (UPGMA) clustering tree. The Bray-Curtis dissimilarity metric, ranging from 0 to 1, provided insight into the degree of microbial community similarity, represented in the heatmap legend as increments of 0.2 up to 0.8 (with 1 excluded). Notably, the UPGMA dendrogram revealed distinct clustering patterns: the control group was entirely separated from patient clusters, while patient subgroups 2 and 3 demonstrated a closer microbial similarity, with subgroup 1 being distinctly separated (Fig. 2B).

Five bacterial species (*Bacillus velezensis*,* Bacillus subtilis*,* Faecalibacterium prausnitzii*,* Blautia sp. SC05B48*,* and Helicobacter apodemus*) were identified as predominant genera across all patient samples. The antibiotic resistance gene (ARG) profiles of these species are presented in Fig. 3A as a heatmap, revealing significant resistance patterns. Notably, *Enterococcus faecalis* was the most abundant microorganism in the healthy control samples, displaying a greater diversity of ARGs compared to patient samples, as shown in Fig. 3B. While typically harmless at low levels, elevated concentrations of *E. faecalis* (> 1 million CFU/mL) can lead to urinary tract infections (UTIs), its reduced presence in patient samples may correspond to the overgrowth of other bacterial species.

The Circos plot (Fig. 4) highlights ampicillin as the antibiotic with minimal ARGs across dominant species, whereas daptomycin shows widespread resistance in both patient and control groups. Pleuromutilin is notable for resistance confined to *Enterococcus* species, with other microorganisms lacking ARGs against this antibiotic. Among dominant bacteria, *Enterococcus* exhibited the highest ARG abundance, followed by elevated ARG presence in *Bacillus* species within patient samples (Fig. 5). These resistance patterns provide insights into potential therapeutic strategies for chronic prostatitis.

**Fig. 6:**
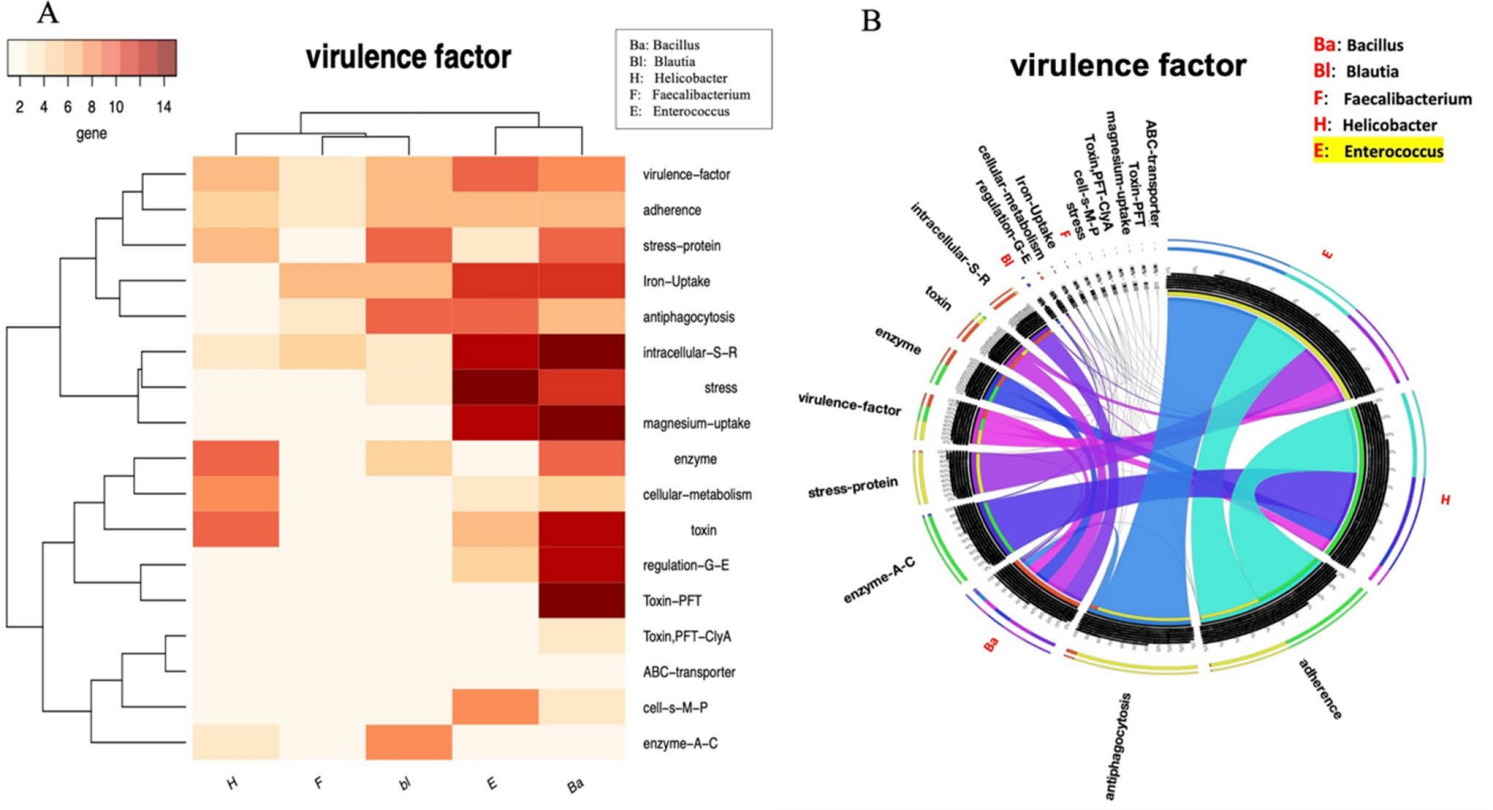
**A**: Heatmap illustrating the distribution of virulence factors (VFs) in the dominant bacterial genera across all groups. The heatmap highlights that Bacillus and Enterococcus genera harbor significantly more virulence factors compared to other microorganisms. This abundance of VFs may explain their antagonistic and competitive relationships with other bacteria. Notably, the higher prevalence of Bacillus bacteria observed in CPPS/CP patient samples in this study could be attributed to this competitive advantage. **B**: Circos plot depicting the virulence factors present in dominant bacterial genera. The visualization emphasizes that adhesion, antiphagocytosis, enzyme-related factors (**A-C**), and stress-protein factors are the major virulence mechanisms associated with the Enterococcus and Helicobacter genera. In contrast, the Bacillus genus exhibits a broader variety of virulence factors, which may confer a competitive edge over other genera. These findings highlight the potential role of VFs in shaping microbial interactions and their relevance in chronic prostatitis pathophysiology

Figure 6 illustrates virulence factors in predominant microorganisms using heatmap (6 A) and Circos (6B) representations. *Bacillus* and *Enterococcus* genera demonstrated higher virulence factor abundance, including adhesion, antiphagocytosis, and stress-protein factors. Notably, *Bacillus* exhibited a broader range of virulence factors, potentially enhancing its survival and colonization in the prostate microbiome. These trends underscore the dynamic interplay between bacterial genera in chronic prostatitis and their implications for microbiome-targeted therapies.

## Discussion

This study found that the *Bacillus* genus constituted a significant portion (29–36%**)** of the microbial community across all patient groups, as confirmed by NCBI and BV-BRC taxonomic analyses. Previous microbiome studies on chronic prostatitis/chronic pelvic pain syndrome (CP/CPPS) have reported altered bacterial communities, with shifts in *Lactobacillus*,* Escherichia*,* Burkholderia cenocepacia*,* Clostridia*,* Proteobacteria*,* Blautia*,* Faecalibacterium*, and *Staphylococcus*. These studies primarily utilized next-generation sequencing (NGS) platforms targeting specific 16 S rRNA regions (e.g., V3 or V4), which, while effective, provided limited taxonomic resolution [26, 27]. In contrast, our study employed nanopore sequencing, enabling whole-genome reads for a more comprehensive analysis of microbial diversity and functional potential. While our findings corroborate previous reports of genera such as *Blautia* and *Faecalibacterium*, we uniquely identified a significantly higher relative abundance of *Bacillus velezensis* across all patient groups compared to healthy controls. This distinct microbial signature, supported by robust effect sizes and confidence intervals, suggests a potentially critical role for *Bacillus velezensis* in inflammation and immune modulation in CP/CPPS. These results emphasize the importance of species-specific investigations, positioning *Bacillus velezensis* as a potential biomarker or therapeutic target.

Although our study had an uneven distribution of participants between CP/CPPS patients (*n* = 58) and healthy controls (*n* = 25), a subgroup analysis of young CP/CPPS patients (*n* = 23) and healthy controls (*n* = 25**)** provided a more balanced comparison. Notably, *Bacillus velezensis* was identified as the dominant bacterial species in the young CP/CPPS group (36%**)**, whereas *Enterococcus faecalis* (52%) predominated in the control group (with *Bacillus velezensis* accounting for 17%**).** These findings reinforce the potential association of Bacillus with CP/CPPS pathogenesis, particularly in younger patients, and underscore the importance of microbial profiling in this context. However, future studies with larger and more evenly distributed cohorts are necessary to confirm these observations and minimize potential biases.

Previous studies have documented *Bacillus velezensis* as part of the human gut microbiome, recognized for its production of various antimicrobial compounds, including surfactin, fengycin, bacillomycin D, and other bioactive metabolites. While these metabolites exhibit antimicrobial properties, they also play a dual role by modulating immune responses, potentially contributing to chronic inflammation.

Surfactin, a membrane-disrupting lipopeptide produced by *Bacillus* species [28], modulates immune responses through its activation of the NF-κB signaling pathway. Upon activation, NF-κB translocates to the nucleus, inducing the transcription of pro-inflammatory cytokines such as IL-6, TNF-α, and IL-1β, which drive immune activation and inflammatory cell infiltration. In chronic prostatitis this activation may amplify cytokine production, promoting immune cell recruitment and perpetuating low-grade inflammation [29].

Beyond cytokine production, NF-κB signaling promotes the upregulation of adhesion molecules such as ICAM-1 and VCAM-1, which facilitate immune cell migration into prostatic tissue [30].This persistent recruitment of macrophages and neutrophils may exacerbate localized inflammation, leading to tissue remodeling and fibrosis, which are hallmarks of chronic prostatitis pathophysiology. Additionally, Surfactin-induced NF-κB activation is closely linked to oxidative stress, with increased reactive oxygen species (ROS) sustaining chronic inflammation and potentially contributing to neurogenic pain sensitization in CP/CPPS [31].

Fengycin, a cyclic lipopeptide produced by *Bacillus* species, plays a multifaceted role in immune modulation by interacting with Toll-like receptors (TLRs), particularly TLR2 and TLR4, which are pivotal in pathogen recognition and the initiation of innate immune responses. Upon binding to these receptors, Fengycin triggers the MyD88-dependent signaling pathway, leading to the activation of nuclear factor-κB (NF-κB) and subsequent transcriptional upregulation of pro-inflammatory cytokines, including IL-6, TNF-α, and IFN-β. This cytokine surge facilitates the recruitment and activation of innate immune cells, such as dendritic cells and macrophages, both of which play essential roles in antigen presentation and sustained inflammatory responses.

In the context of chronic prostatitis/chronic pelvic pain syndrome (CP/CPPS), the continuous stimulation of TLRs by Fengycin could promote persistent immune activation, leading to prolonged inflammation and localized tissue damage. This chronic immune stimulation may also influence adaptive immune responses, as TLR engagement has been linked to the upregulation of immune checkpoint molecules such as programmed cell death protein 1 (PD-1) and T-cell immunoglobulin and mucin-domain containing-3 (TIM-3). These checkpoint molecules, while essential for maintaining immune homeostasis, can become dysregulated in chronic inflammatory conditions, impairing T-cell effector functions and contributing to immune exhaustion [32, 33].

This dysregulation is particularly relevant in CP/CPPS, where unresolved inflammation is a defining feature of the disease. The interplay between TLR activation, sustained cytokine production, and immune checkpoint dysregulation may perpetuate a vicious cycle of inflammation, preventing immune resolution and facilitating chronic disease progression. Given Fengycin’s dual role in both pathogen defense and immune activation, further studies are needed to elucidate whether targeting TLR-mediated pathways could mitigate pro-inflammatory effects while preserving host defense mechanisms. These findings emphasize the need for precision-targeted therapies that can modulate TLR-driven immune responses to prevent chronic inflammation in CP/CPPS while maintaining microbial homeostasis.

Bacillomycin D, a polyketide antibiotic produced by *Bacillus velezensis*, plays a dual role as both an antimicrobial agent and an immune modulator. While it exhibits potent antifungal and antibacterial properties, its interaction with host cells induces oxidative stress, triggering inflammatory pathways. Oxidative stress activates the Nrf2 (Nuclear factor erythroid 2-related factor 2) pathway, a key regulator of antioxidant defenses, and initiates MAPK (Mitogen-Activated Protein Kinase) signaling cascades, including JNK, p38 MAPK, and ERK. While Nrf2 activation mitigates oxidative damage through antioxidant gene transcription, prolonged oxidative stress can sustain MAPK activation, leading to persistent inflammation by promoting the production of pro-inflammatory cytokines, such as IL-6 and TNF-α, and enhancing immune cell infiltration into inflamed tissues. In the context of CP/CPPS, chronic oxidative stress and sustained MAPK activation may exacerbate immune cell recruitment, disrupt tissue homeostasis, and contribute to prostate inflammation and fibrosis. Moreover, oxidative stress-mediated immune checkpoint dysregulation can impair resolution of inflammation, prolonging the chronic immune activation state characteristic of CP/CPPS. These findings suggest that Bacillomycin D, while beneficial as an antimicrobial agent, may inadvertently contribute to chronic inflammation and immune dysregulation, particularly when Bacillus species are dominant in the microbiome.

Collectively, *Bacillus*-derived metabolites, including surfactin, fengycin, and bacillomycin D, influence multiple immune signaling pathways through NF-κB, TLRs, MAPK, and Nrf2 activation. Their effects orchestrate a pro-inflammatory environment, driving chronic inflammation, immune cell recruitment, cytokine production, and immune checkpoint dysregulation, all of which are hallmarks of CP/CPPS pathology [34]. Given the dominance of *Bacillus velezensis* in CP/CPPS patients, our findings highlight the need for caution when considering these metabolites in therapeutic applications, as their immune-modulating properties may exacerbate inflammation rather than alleviate it. These results underscore the importance of thoroughly evaluating the therapeutic implications of *Bacillus* metabolites, particularly in chronic inflammatory conditions such as CP/CPPS, where microbial dysbiosis and persistent inflammation are key disease-driving factors. These metabolites highlight the dual role of *Bacillus velezensis* as both an inflammatory and competitive agent within the microbiome. Maintaining microbiome composition and diversity is essential for host health, and our findings reveal a strong antagonistic relationship between *Bacillus* and *Enterococcus* species in the urinary microbiome.

As competitive agents, *Bacillus* species secrete antimicrobial compounds (e.g., surfactins, bacillomycin, and fengycin) that inhibit pathogens and promote microbiome stability. However, as noted earlier, these same metabolites also modulate immune responses, stimulating cytokine production and potentially exacerbating local inflammation in CP/CPPS patients. In this context, immune modulation refers to the ability of *Bacillus* metabolites (e.g., surfactins) to induce pro-inflammatory cytokines (IL-6, TNF-α), which may contribute to persistent inflammation in CP/CPPS. Similarly, virulence factors, including adhesion proteins, stress-response enzymes, and biofilm-associated structures, enhance *Bacillus* species’ survival, competition, and persistence within the microbiome. These mechanisms not only establish *Bacillus* as a dominant species but may also sustain inflammatory conditions, further complicating CP/CPPS pathogenesis.

The dual role of *Bacillus velezensis*—as both a microbial competitor and a pro-inflammatory agent—underscores the complexity of microbiome interactions in CP/CPPS. Future studies should investigate how these mechanisms drive disease progression and explore whether targeted interventions can mitigate the inflammatory effects of *Bacillus*metabolites while preserving microbiome balance.

Moreover, *Bacillus velezensis* produces a diverse array of metabolites, including protease and cellulase enzymes, which interact with the host in complex ways. Proteases can trigger leukocyte infiltration and exacerbate immune responses, potentially leading to inflammatory reactions in the urinary tract [35]. This aligns with previous studies that reported elevated leukocyte levels in CP/CPPS patients compared to healthy controls [9], further supporting the role of Bacillus-derived metabolites in sustaining chronic inflammation.

Additionally, biofilm formation by *B. velezensis* may contribute to infection persistence in CP/CPPS. Biofilms provide a protective barrier, allowing bacteria to evade host immune responses and develop increased resistance to antimicrobial treatments. Prior research on *B. velezensis* FZB42 has identified biofilm-associated gene clusters, underscoring the species’ adaptability and resilience in hostile environments [36, 37]. This suggests that biofilm production may serve as a key survival mechanism for *Bacillus* in the urinary tract, potentially perpetuating chronic inflammation and treatment resistance in CP/CPPS patients.

Together, these findings provide further evidence of the complex microbial dynamics in CP/CPPS. The observed increase in *Bacillus* species alongside a decrease in *Enterococcus* species may contribute to symptom severity and chronic disease progression. Given the antimicrobial properties and immune-modulating capabilities of *Bacillus velezensis*, precision antimicrobial strategies should aim to target pathogenic strains while preserving microbial balance, preventing unintended disruptions to the urinary microbiome and improving long-term clinical outcomes.

In addition, our study provides insights into the distinct microbial landscape and immune dynamics of CP/CPPS, particularly among younger patients, who exhibited greater microbial diversity compared to older individuals. Aging is associated with hormonal changes, including declining testosterone and increasing estrogen levels, which may disrupt the prostate and urinary tract microbiome, potentially contributing to chronic prostatitis. Moreover, fluctuations in dihydrotestosterone (DHT), a hormone essential for prostate health, could further influence microbial populations [38].

Besides hormonal changes, lifestyle factors such as diet, sedentary behavior, hydration, hygiene, and sexual health practices significantly impact microbial composition. Notably, an active sexual life, particularly with multiple partners, may contribute to distinct microbial signatures in the urinary microbiome of younger individuals. Consistent with previous studies, our findings revealed significant differences in microbiome diversity and composition between younger and older CP/CPPS patients, potentially explaining why CPPS is the most frequent urological diagnosis in men under 50 years old [39].

These results highlight the interplay between age-related hormonal shifts, lifestyle influences, and microbial diversity, reinforcing the notion that while aging and hormonal changes contribute to CP/CPPS, lifestyle factors—especially those linked to sexual health and hygiene**—**may play a more significant role in disease onset and severity [38]. The high prevalence of CPPS in younger individuals further supports this hypothesis, underscoring the need for age-specific approaches to understanding and managing the condition.

The microbial shifts observed in CP/CPPS patients, particularly the reduced presence of *Enterococcus faecalis*, indicate a disruption in urinary microbiome balance. *E. faecalis* plays a stabilizing role by preventing the overgrowth of potentially pathogenic bacteria; thus, its depletion may favor species associated with chronic inflammation. Restoring microbial homeostasis could serve as a therapeutic target for alleviating symptoms and mitigating inflammation in CP/CPPS patients. In this context, our bioinformatic analysis of dominant bacterial genomes further revealed significant antibiotic resistance gene (ARG) patterns, emphasizing the necessity for targeted therapeutic strategies. Notably, *Bacillus velezensis* and *Bacillus subtilis* harbored a higher prevalence of ARGs compared to *Faecalibacterium prausnitzii*, *Blautia sp. SC05B48*, and *Helicobacter apodemus*. *Enterococcus* species demonstrated strong resistance to pleuromutilin and vancomycin, while other dominant bacterial genera exhibited limited resistance to these antibiotics. Of particular note, pleuromutilin, including its synthetic derivative ferroptocide (FTC), has shown promise as a therapeutic option in prostate cancer treatment [40]. Vancomycin, administered intravenously, exhibits effective penetration into prostate tissue, making it a viable candidate for prostatitis treatment by reducing inflammation and promoting tissue repair [41]. While ampicillin remains broadly effective against various bacteria, its potential adverse effects on beneficial species like *Enterococcus* may limit its suitability for CP/CPPS treatment. Reduced resistance to ampicillin among dominant bacterial strains suggests its possible efficacy in specific cases [42]. However, notable resistance to daptomycin, particularly in *Enterococcus faecalis*, *Bacillus velezensis*, and *Bacillus subtilis*, presents a significant therapeutic challenge. Addressing these resistance patterns in conjunction with bacterial virulence mechanisms could inform advanced treatment strategies. For instance, targeting adhesion factors and enzymatic activity in *Bacillus* or stress-protein factors in *Enterococcus* may help disrupt bacterial persistence and alleviate CP/CPPS symptoms.

These findings contribute to a deeper understanding of pathogen-specific resistance patterns and highlight the potential of precision medicine approaches in combating chronic prostatitis. Although bioinformatics tools provided robust analyses and identified potential antibiotic candidates, experimental and clinical validation of these resistance mechanisms remain essential, as they were beyond the scope of this study. Further investigation into single nucleotide polymorphisms (SNPs) influencing antibiotic resistance in bacteria is essential for refining therapeutic strategies and optimizing patient outcomes. As mentioned earlier, bioinformatic analyses can detect the presence of antibiotic resistance genes (ARGs) within dominant bacterial genomes, helping determine whether these genes functionally confer resistance or remain inactive. SNPs within these genes or their regulatory regions may alter their function, potentially enhancing resistance mechanisms or, conversely, reducing susceptibility to antibiotics [43]. A deeper SNP analysis could improve predictions of resistance phenotypes, guiding clinicians toward more targeted antibiotic choices while avoiding ineffective treatments. Additionally, this approach may aid in uncovering novel resistance mechanisms, offering new therapeutic targets for CP/CPPS.

Despite the valuable insights gained from this study, several limitations should be acknowledged. While advanced bioinformatics platforms (BV-BRC, Galaxy) provided robust microbial composition and resistance gene analyses, experimental validation was beyond the study’s scope. It remains unclear whether the identified antibiotic resistance genes (ARGs) are functionally active, emphasizing the need for functional assays in future studies. Additionally, the cross-sectional design captures only a single time point, limiting conclusions on causality and disease progression. Longitudinal studies are needed to track microbiome evolution and assess its impact on symptom severity and treatment response over time.

The relatively small sample size and limited geographic diversity may also impact the generalizability of these findings. Although strict inclusion criteria minimized confounding factors (e.g., recent antibiotic use), expanding the cohort across multiple regions would provide a broader understanding of microbial variations in CP/CPPS. Moreover, while probiotic therapy was discussed as a potential treatment, no experimental validation was performed to confirm its efficacy or safety. Further research should evaluate the clinical impact of probiotic supplementation and its potential to improve microbiome stability without unintended disruptions.

Given the intricate interplay of microbial dynamics in CP/CPPS, metagenomic profiling should be incorporated into clinical decision-making to guide precision-targeted antimicrobial therapy. Since lifestyle factors influence microbiome composition, assessing individual microbial diversity is essential for identifying dominant bacterial species and optimizing therapeutic approaches. Our study identified *Bacillus velezensis* as the predominant species in CP/CPPS patients, underscoring the importance of careful antibiotic selection. Vancomycin and pleuromutilin appear to be effective against Bacillus while preserving beneficial *Enterococcus* species. However, maintaining balanced microbial populations is critical to prevent potential secondary infections such as urinary tract infections (UTIs). Regular monitoring of *Bacillus* resistance patterns is necessary to guide antibiotic selection and ensure long-term efficacy. Beyond antibiotic therapy, microbiome modulation strategies may provide additional therapeutic benefits. Probiotics containing *Enterococcus* could support microbial homeostasis during or after treatment, but their administration must be carefully regulated to prevent unintended shifts in microbial dominance. Lifestyle interventions, such as adequate hydration, proper nutrition, and good hygiene, can also help stabilize the microbiome and reduce the likelihood of CP/CPPS recurrence.

Furthermore, our findings highlight the need for caution when considering *Bacillus velezensis* as a probiotic agent due to its pro-inflammatory properties and potential role in CP/CPPS pathogenesis. While Bacillus-derived metabolites can suppress pathogens, their immune-modulating effects may contribute to chronic inflammation in susceptible individuals. Future studies should investigate these mechanisms further to refine microbiome-targeted therapies and develop precision medicine approaches for CP/CPPS.

Collectively, these findings emphasize the importance of microbiome-based therapeutic strategies in CP/CPPS management. Future research should explore SNP-based resistance mechanisms, precision probiotics, and microbiome-targeted interventions to develop individualized treatment options that address both microbial imbalances and chronic inflammation in CP/CPPS patients.

## Conclusion

This study underscores the role of microbial dysbiosis in CP/CPPS pathogenesis, particularly the dominance of Bacillus velezensis and its association with pro-inflammatory and competitive microbial interactions. The identification of antibiotic resistance genes and virulence factors in dominant bacterial species further supports the need for microbiome-based therapeutic strategies. While pleuromutilin and vancomycin emerged as potential targeted antibiotics, careful consideration of microbiome balance is essential to prevent unintended disruptions.

Given the distinct microbial diversity observed in younger CP/CPPS patients, future studies should explore the interplay between microbial shifts, immune responses, and disease progression across different age groups. Integrating metagenomic analysis into clinical practice could enhance personalized treatment approaches, optimizing antibiotic selection while preserving beneficial microbiota. These findings provide a foundation for precision medicine in CP/CPPS management, highlighting the need for further functional validation and longitudinal studies to refine microbiome-targeted therapeutic interventions.

## Electronic supplementary material

Below is the link to the electronic supplementary material.

Supplementary Material 1

## Data Availability

Sequence data that support the findings of this study is provided within supplementary information files.
