# Supplementary material for: Bioinformatic strategies in metagenomics of chronic prostatitis
Source: World J Urol. 2025 Mar 26;43(1):188. doi: 10.1007/s00345-025-05514-7 (PMC11947071; doi:10.1007/s00345-025-05514-7)

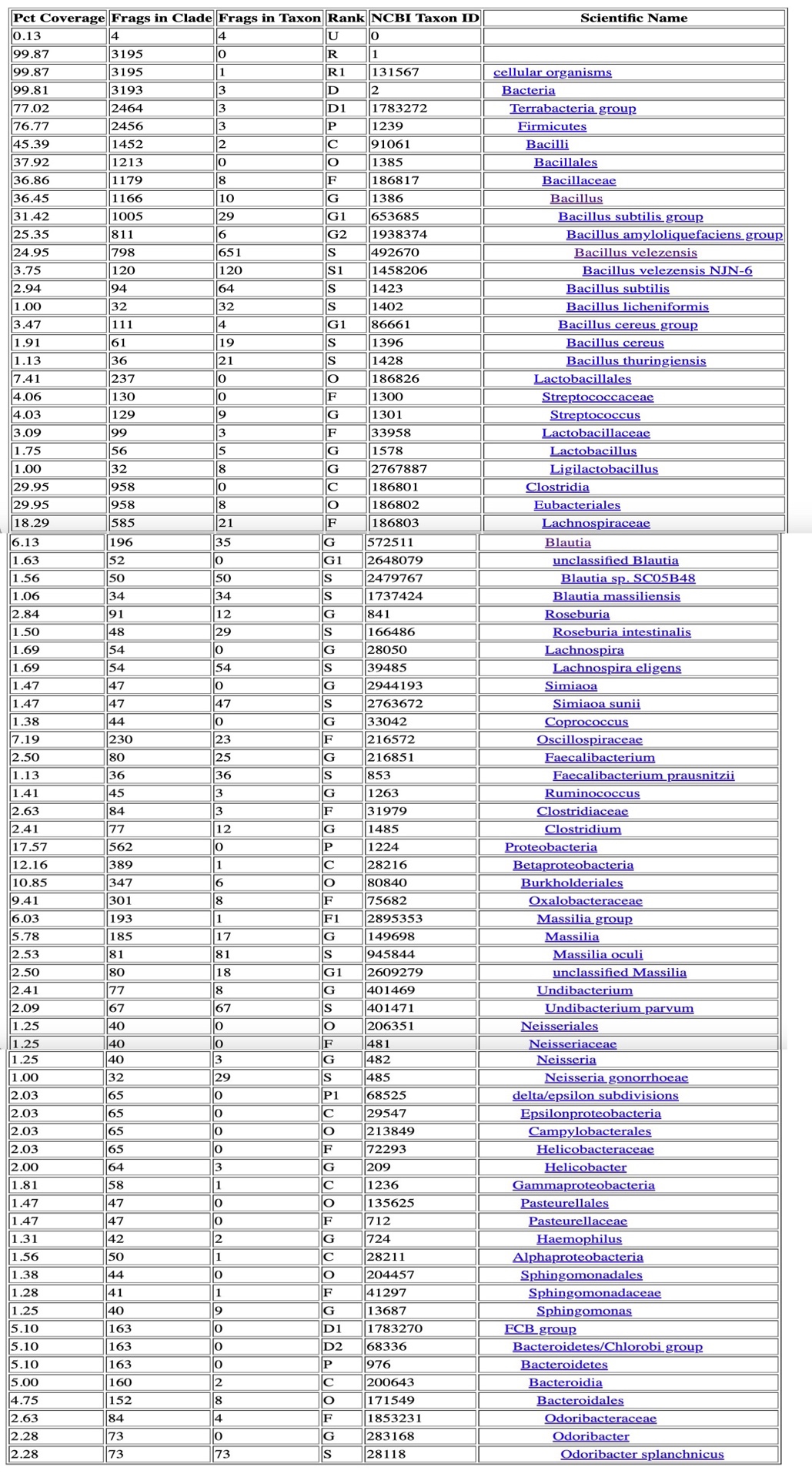


*Table 1: Taxonomic Classification Report for group 1. Input data: Single-end Libraries.*

Table 2: Taxonomic Classification Report for group 2. Input data: Single-end Libraries.


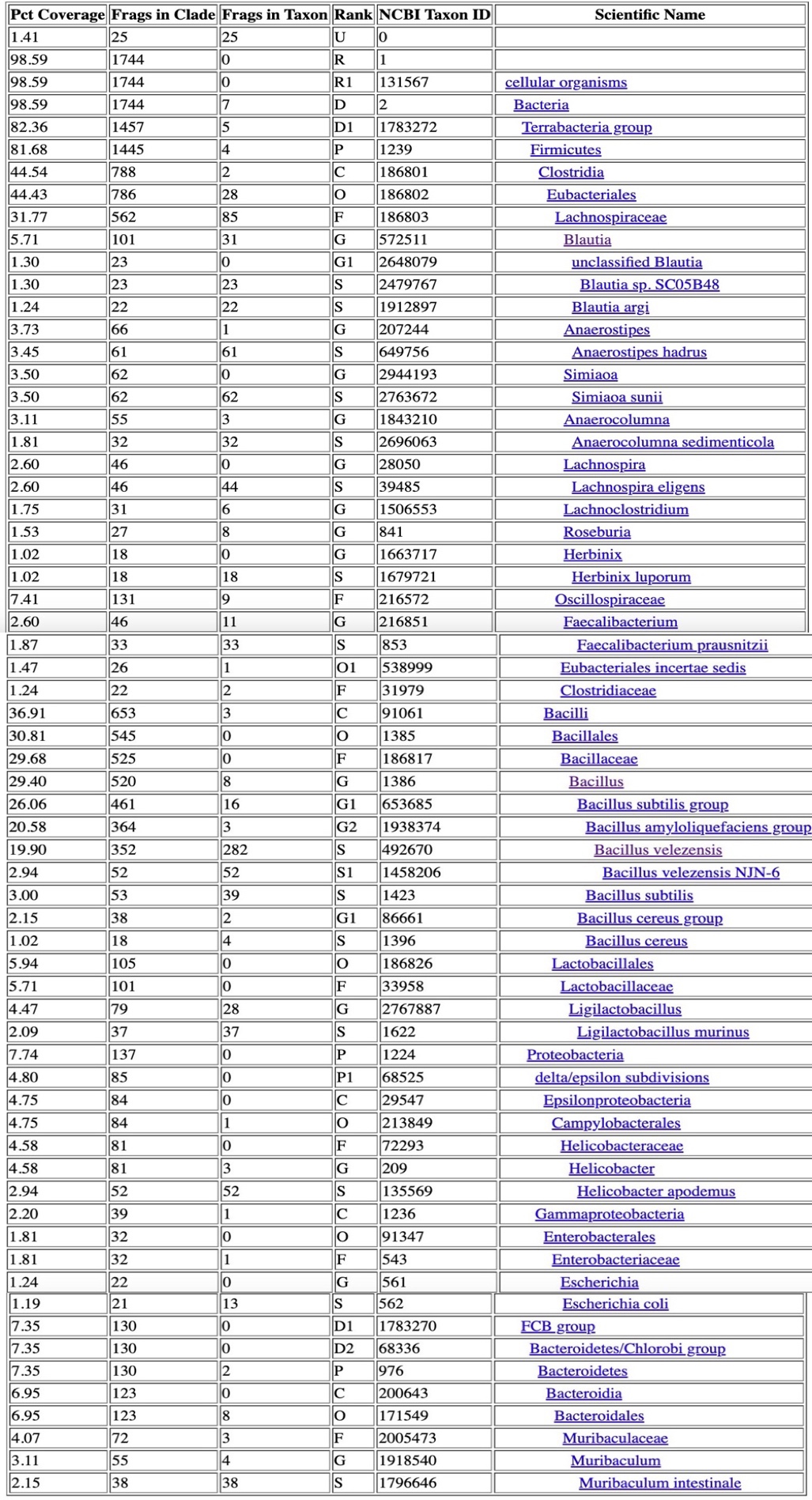


Table 3: Taxonomic Classification Report for group 3. Input data: Single-end Libraries.


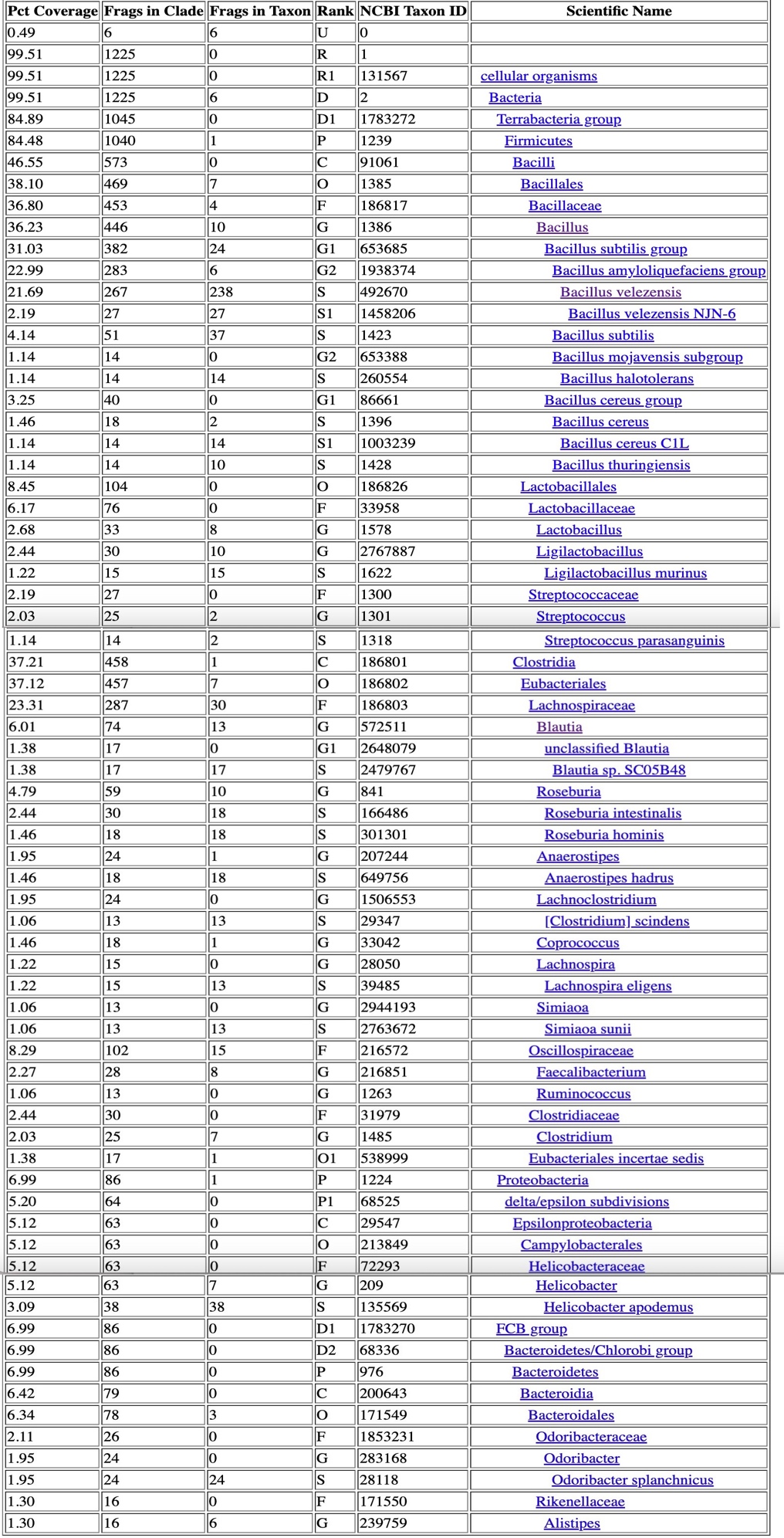


Table 4: Taxonomic Classification Report for control group. Input data: Single-end Libraries.


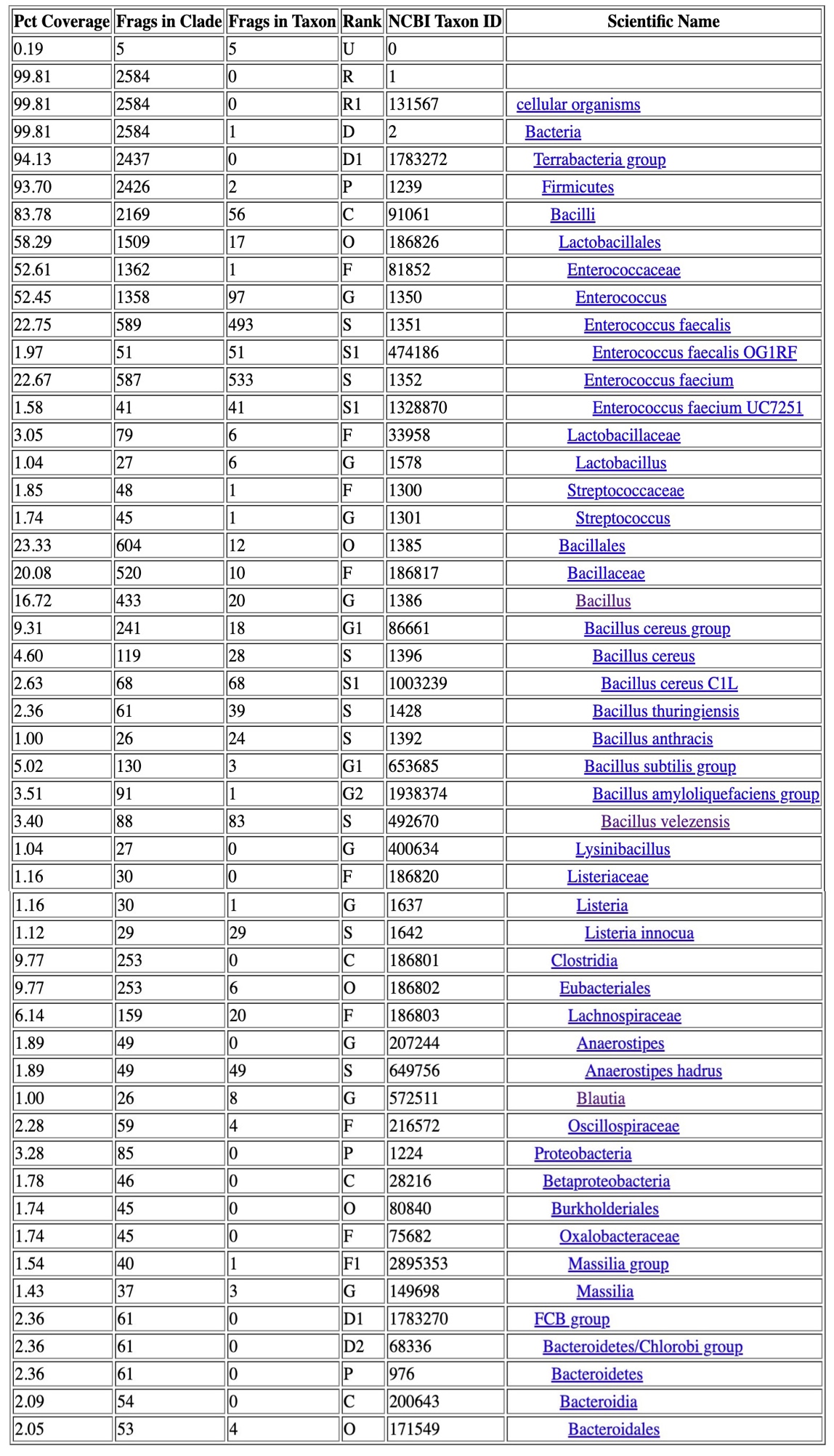

Supplement: Supplementary file 1 — Supplementary Material 1 [file 345_2025_5514_MOESM1_ESM.docx]
